# Yuri B. Yurov (1951-2017)

**DOI:** 10.1186/s13039-018-0383-3

**Published:** 2018-06-13

**Authors:** Ivan Y. Iourov, Svetlana G. Vorsanova

**Affiliations:** 1 Laboratory of Molecular Genetics and Cytogenomics of the Brain, Mental Health Research Center, Zagorodnoe shosse 2/16, 117152 Moscow, Russia; 2 Veltischev Research and Clinical Institute for Pediatrics of the Pirogov Russian National Research Medical University, Ministry of Health of Russian Federation, Moscow, 125412 Russia

*— It will take people ten years to forget!*

*— It will indeed be honored across two epochs!*

*Marina I. Tsvetaeva*

**Fig. 1:**
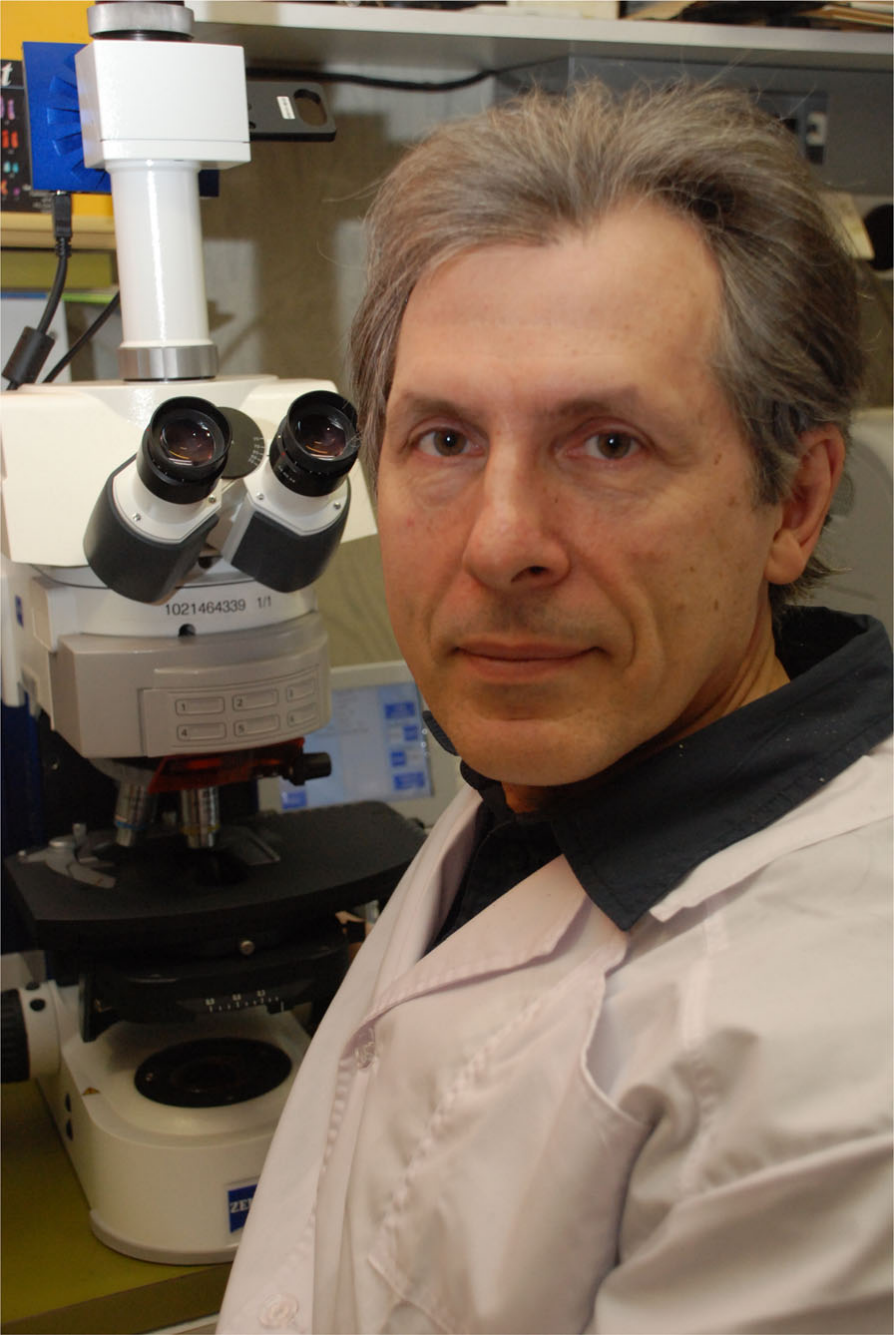
Yuri B. Yurov (1951-2017)

On 12 December, 2017, Professor Yuri Borisovich Yurov (Fig. 1) passed away peacefully at the Federal Scientific Clinical Center for Resuscitation and Rehabilitation, a renowned resuscitation/rehabilitation institute located not far from Moscow. The tragic, albeit inevitable, ending of Yuri’s courageous fight against a devastating cancer has suddenly stricken all his colleagues and friends. Our deepest sorrow has paralyzed our capacity to do anything, leading us to recognize the vital need for reevaluating Yuri’s scientific and personal legacy.

Yuri’s life is an example of unselfish devotion to bioscience. His contribution to genetics and practical medicine is hard to estimate. It is truly immense! Since Yuri’s personality can be defined using such epithets as “bright intelligence”, “overwhelming kindness”, and, especially, “extreme modesty”, it is not surprising that he was always skeptical about “aggressive public relations efforts” to advance careers and theories, which are so popular in modern biomedicine. Thus, one should not be surprised about the lack of annoyingly large lists of his awards, honors, commitments, etc. Certainly, Yuri’s research was awarded local and international prizes and grants from time to time. These, however, are a poor reward for his tremendous endeavors to organize, undertake, and present such a groundbreaking body of work. To redress this historical injustice, we decided to describe Yuri’s life in a biographical review instead of a traditional obituary limited to a short rueful notification. We do hope that our effort to share the experience of being alongside this brilliant researcher and bright person gives a succinct understanding of Yuri’s profound, albeit non-explicit, impact on biomedicine and neuroscience.

Yuri was born on 11 December, 1951, in Zhukovsky, a city near Moscow known as a home to several major research institutes involved in designing aircraft, to a family of an engineer-researcher. While graduating from school, he chose biology as a field of further higher education. Today, we know that it was a good choice. His admission to the Faculty of Biology at Lomonosov Moscow State University was associated with a story that picturesquely exposes the essence of Yuri’s personality. The initial results of the matriculation examination did not allow his admission to the biological faculty of Lomonosov Moscow State University, but still allowed him to enter the Faculty of Biology at Moscow State Pedagogical Institute. Later on, he unintentionally told his father that he is “100% sure” to pass the matriculation examination at Lomonosov Moscow State University. His father decided to appeal to the university in this regard, which revealed that Yuri was right. As a result, he was admitted to the faculty of biology at Lomonosov Moscow State University. There, he made the ultimate decision to become a researcher in genetics.

In the beginning, Yuri studied DNA replication [1]. Initially, his research was performed at Kurchatov’s Institute of Moscow with Dr. Evgenii Ananiev, a recognized Russian geneticist who contributed to the research of mobile genetic elements and plant chromosomes [2, 3]. He pursued the research of DNA replication at Institute of Medical Genetics (Academy of Medical Sciences of the USSR, Moscow) and made appreciable progress thereof [4–10]. In 1977, Yuri defended his Ph.D-thesis “Replicational organization of chromosomal DNA in cultured normal and abnormal cells of humans and animals”. However, he was obliged to cease studying DNA replication. As we previously implied, Yuri was completely disinterested in backroom intrigues and local politics. This was not the case for the majority of group leaders and administration representatives at the medical genetics institutes at the end of seventies and beginning of the eighties. Fortunately, psychiatric genetics was a research focus of the All-Union Mental Health Research Center (Academy of Medical Sciences of the USSR, Moscow), where Yuri was invited to develop new techniques for studying the human genome at the chromosomal level in brain disorders by Professor Marat E. Vartanian, who had then become the head of this center. Henceforth, his research career would be tightly linked to Mental Health Research Center, where he successfully completed his doctoral dissertation for receiving the degree of Doctor of Science (the highest academic degree achieved through defending a thesis significantly more voluminous than a canonical Ph.D thesis) — “Molecular cytogenetics of heterochromatic regions in the human genome” — and became the head of the Cytogenetics Laboratory (laboratory of cytogenetics and genomics of mental disorders).

So began Yuri’s molecular cytogenetic odyssey. He was among the first researchers in the world to develop a technique of in situ hybridization with cloned DNA sequences for the analysis of human chromosomes [11]. Almost immediately, the results of these developments were found applicable for molecular cytogenetic diagnosis of chromosomal abnormalities. Unfortunately, the article describing these applications was published two years later after the submission along with other articles reporting similar results [12]. His molecular cytogenetic studies were based on a part of his immense research activity referred to molecular, cytogenetic and evolutionary analysis of alpha satellite DNA and constitutive heterochromatin [13–19]. Evolutionary molecular (cyto)genetic studies have also underlined long-standing friendship and collaboration with Professor Gérard Roizès (Institut de Biologie, Montpellier, France) [20], with whose team (équipe) he worked for several years. During this period, he had a family tragedy. His stepson — Dr. Ilia V. Soloviev, a prodigious young researcher and a pioneer of molecular cytogenetics and cytogenetic genome research, whose brilliant work and original ideas still form research directions in our labs — tragically passed away. This sorrow led to re-assessing life priorities. More intense research was undertaken to decrease the suffering.

Inspired by the first success of his molecular cytogenetic research and diagnosis [21–25], Yuri together with his closest colleagues undertook further studies to increase the efficiency and scope of molecular cytogenetics. Thus, a series of (ultra) rapid fluorescence in situ hybridization (FISH) protocols was proposed [26, 27]. One rapid protocol, based on microwave activation [26], was developed 10-15 years before the introduction of such approaches to diagnostic and research practice. Additionally, FISH-based molecular cytogenetic techniques for the identification of marker chromosomes were proposed [28–30]. At that time, the essential issue of Yuri’s research was the creation of an original DNA probe collection [16, 17, 31], which formed a firm basis for almost all our further studies and developments in molecular cytogenetics. For instance, interphase detection of chromosome 21 aneuploidy for prenatal diagnosis was significantly improved [27, 32]. Multicolor interphase FISH with centromeric (chromosome-enumeration) DNA probes was the next major breakthrough of research headed by Yuri [33]. This technique is still very popular and is actually the most direct way to analyze numerical chromosomal changes in large cellular populations at single-cell level. It was also found applicable for studying sperm providing new opportunities for reproductive genetics [34]. These studies can be designated as the main basis for the first generation interphase (molecular) cytogenetics developments allowing the analysis of chromosomal loci or ambiguous/amorphous chromosome territories (chromosomal parts’ territories) without an integral view of the whole interphase chromosomes.

Another layer of Yuri’s research is represented by studying X-linked diseases [35–37], more precisely, Rett syndrome. The studies of Rett syndrome performed with his participation were global [38, 39]. These included the confirmation of an X-linked nature of the syndrome [40–42], mutational analysis in Russian Rett syndrome cohorts [43], uncovering pathoepigenetic mechanisms specific for Rett syndrome (i.e. alterations to chromosomal DNA replication, X-linked bi-allelic expression, parent-of-origin-like effects on X chromosome inactivation) [40–42, 44–46], and discovering the microdeletion nature of *MECP2-*mutation negative Rett syndrome cases [47, 48]. The success of Rett syndrome studies culminated in 2016, when the VIII World Rett Syndrome Congress (scientifically organized with active Yuri’s participation) was held in Kazan, Russia. The congress highlighted true success and recognition of Rett syndrome research in Russia, which is, in turn, the result of Yuri’s efforts. Regretfully, due to numerous unexpected problems, we have not still published the article describing this congress. Nonetheless, we await this article to be published in *Molecular Cytogenetics* soon*.*

Yuri’s original collection of DNA probes was found useful in numerous studies performed by our labs and his collaborators all over the world. These studies included, but were not limited to, analyses of chromosomal abnormalities and instability (mosaicism) in a variety of human tissues [49–52], mapping breakpoints of structural chromosome rearrangements [52–55], discovering the role of chromosomal mosaicism (mosaic aneuploidy and polyploidy) in spontaneous abortion [56–60]. However, there was a real need to widen the spectrum of molecular cytogenetic techniques, especially for studying interphase chromosomes and genomic (chromosomal) variations at single-cell level in different tissues [61–65]. In this context, it is to mention one of the major theoretical contributions to bioscience made by Yuri.

It has been postulated without any evidence that all cells of an organism possess identical genomes. Taking into account simply the amount of mitoses needed to generate the required amount of cells of an organism, we inevitably come to a conclusion that these postulates are nonsense. Although the scale of mosaicism in somatic tissues is to be determined more thoroughly, one had to admit the existence of overlooked cellular fractions featured by unshared genomes [66]. Regardless of being presented as an encyclopedic knowledge [67], intercellular/somatic genomic variations were more-or-less recognized as an important mechanism for interindividual diversity in health and disease during the last few years only. Moreover, we had proposed a neurocytogenetic hypothesis suggesting that genetic mechanisms of brain diseases are more likely to be related to cellular populations with abnormal genomes primarily affecting the brain. In other words, Yuri insisted that we should perform genetic analysis of the brain to uncover the mechanisms of central nervous system diseases [68]. As one can guess, the idea encountered serious resistance from skeptical ignorance to aggressive denial. It is rare for a researcher to prefer to break a tradition to the detriment of its “publicability” and funding opportunities. When the overwhelming majority of studies in psychiatric (medical) genetics are made using DNA isolated from blood, it is naïve to think that such ideas may be accepted quietly. Yuri did not care much about it. He preferred creating trends to following trends. Interestingly, this approach to brain diseases is quite popular in the latest neuroscience literature, unfortunately often without appropriate references to the original theoretical articles.

To succeed in studying somatic genome variations in the brain, new molecular cytogenetic techniques were strongly required. Using positive experience in developing original computer-assisted analysis of FISH results [69], we elaborated a quantitative FISH protocol [70, 71]. The latter was found applicable for analysis of chromosomal heteromorphisms/pericentromeric regions (i.e. identification of parental origins of homologous chromosomes with efficiency comparable to PCR-based methods) [72], distinguishing between interphase chromosome loci pairing or associations and chromosomal loss [73], and determining specific genome architecture within human interphase nuclei [74]. Still, the possibility to see an interphase chromosome in its integrity was not available. To offer the opportunity to see a banded interphase chromosome, we took advantage of a collaboration with Professor Thomas Liehr (Jena, Germany) for developing a new method for analysis of interphase chromosomes in their integrity, entitled interphase chromosome-specific multicolor banding (ICS-MCB) [75–78]. Finally, there was a lack of protocols for obtaining cellular suspensions from postmortem brain specimens applicable for specific FISH-based approaches to single-cell interphase chromosome analysis. The problem was soon solved [79] and became a continuously applicable method for human molecular neurocytogenetics [80]. The results of these interphase cytogenetic achievements were summarized in quite a highly cited (for non-canonical cytogenetics) review [81] and in quite a widely read book [82].

Yuri’s idea about the link between pathogenesis of psychiatric, neurodevelopmental, and neurodegenerative diseases and genetic pathology exclusively affecting the central nervous system required the knowledge concerning the background rate of sporadic (chromosomal) mutations in the unaffected brain. Under his leadership with the valuable help of Professor Sergei Kutsev, who provided the unique specimens, we performed analysis of chromosomes in the developing human brain [83–86]. As a result, it had been sensationally reported that the overall percentage of aneuploid cells is 30–35% in the developing brain indicating aneuploidization and developmental chromosomal instability to be an additional pathological mechanism for neuronal genome diversification at molecular and cellular levels [86]. In the unaffected postnatal brain, these rates are significantly lower [75, 84, 87, 88].Therefore, Yuri’s hypothesis was supported at this stage.

Schizophrenia was the first disease considered in the neurocytogenetic context. Actually, it was the first neurocytogenetic analysis in human with the special attention to mosaicism confined to the brain showing chromosomal abnormalities/instability causative for mental illness [89]. This is mainly due to the fortunate availability of the Mental Health Research Center collection of postmortem brain specimens managed by Dr. Viktor Vostrikov and Professor Natalia Uranova (Mental Health Research Center, Moscow, Russia). Interestingly, somatic mosaicism, even in a wider sense (i.e. unconfined to a tissue), was not a focus of psychiatric genetics research at all at that time [90]. During the next years, Yuri’s neurocytogenetic research of schizophrenia was successful. We were able to demonstrate the disease association with chromosome 1-specific aneuploidy (instability) and to show the involvement of brain-specific low-level post-zygotic aneuploidy in schizophrenia and comorbid psychiatric disorders [91–94].

Neurodegenerative diseases were another focus of Yuri’s neurocytogenetic research [87]. First, Alzheimer’s disease was found to be associated with aneuploidy confined to the brain. Moreover, it marked the end of debates about parallels between Alzheimer’s disease and Down syndrome. Consequently, it was postulated that Alzheimer’s disease is associated with chromosome 21-specific aneuploidy and instability in the brain, but it is not a subtype of Down syndrome (trisomy 21) [87, 95, 96]. This devastating neurodegenerative disease is likely to result from a complex mechanism involving abortive cell cycle re-entry, replication stress in postmitotic neural cells, genome instability, and deposition of amyloid beta-peptide [97]. Furthermore, additional studies showed a link between cytogenetic markers of aging and Alzheimer’s disease pathogenesis [98]. Accordingly, numerous hypotheses about mechanisms for Alzheimer’s disease were merged together to form the Alzheimer’s disease pathogenetic cascade.

The support, provided by Ataxia Telangiectasia Children’s Project, significantly helped Yuri to pursue his neurocytogenetic research of neurodegenerative diseases. Yuri focused on an intriguing paradox of ataxia telangiectasia: exclusive cerebellar neurodegeneration [99]. This neurodegenerative disease was found to exhibit chromosome instability in the brain [87]. In addition, ataxia telangiectasia also demonstrated a link between area- and chromosome-specific instability and neurodegeneration [100]. These findings were then suggested to be a likely basis for the therapeutic interventions in neurodegenerative diseases mediated by chromosome instability [101]. Thus, the first evidence that genome/chromosome instability is able to produce neurodegeneration, whereas it is generally assumed to be associated with cancer, was provided.

Parallelly, somatic (cyto)genomic variations were studied in autism. As a result, it was discovered that somatic mosaicism (aneuploidy) is a genetic risk factor for autism [102]. Furthermore, these studies demonstrated an unprecedentedly high prevalence of chromosomal heteromorphisms in autistic children [103, 104]. So far, these two types of genomic variations seem to be the most common ones in this pervasive neurodevelopmental disorder. Theoretically, these data were applicable for explaining the male-to-female ratio in autism [105]. Furthermore, chromosomal heteromorphisms and instability were then shown to co-segregate with mental illness in autistic pedigrees [106, 107]. These studies were followed by neurocytogenetic analysis of the autistic brain. We do hope to present the results in the nearest future.

This area of Yuri’s research allowed proposing a “global mosaicism pathway” for human intercellular/interindividual diversity and disease pathogenesis throughout the ontogeny [108]. Interestingly, it was associated not only with aneuploidy, but also with structural chromosome abnormalities (i.e. dynamic mosaicism, tissue- (or sub-tissue) specific mosaicism and local tissue-specific increase of chromosome instability [100, 109]. Furthermore, this “global mosaicism pathway” highlighted developmental chromosome instability as a possible cause of cancer in early childhood [110]. Finally, the pathway explained cell senescence and aging of tissues composed of postmitotic cells through the accumulation of somatic (chromosomal) mutations [111]. All these experimental and theoretical analyses underlined the basis of molecular cytogenomics, which aggregated and correlated data on heritable and non-heritable (somatic mosaicism) genomic variations [112]. It is noteworthy that the term “cytogenomics” was suggested by Yuri ten years before it started to be widely used in similar contexts. This enormous body of research culminated in publishing a special issue on somatic genome variations (mosaicism) in *Current Genomics* [113]. In that issue, Yuri and colleagues provided three main directions of studying somatic genome variations: somatic mosaicism’s role in health and disease [114], somatic genome variations in the ontogenetic context [115], and diagnostic issues of studying somatic genome variability [116]. The paradigm of somatic genomics was then repeatedly postulated indicating the global contribution of genome/chromosome instability to brain diseases [117–120]. Finally, somatic genomic theory suggested a new generalized pathway, linking germline/heritable genome variations, somatic mosaicism and genetic-environmental interactions [121]. Yuri’s research of somatic genome variations is another example of his major contribution to bioscience. He did succeed to persuade biomedical researchers that genomes of somatic cells are not identical. So, the human is not a parody of a huge unicellular organism anymore.

Summarizing the developments in single-cell biology and data on somatic mosaicism, we immediately found that systems biology approaches are required for a successful molecular diagnosis and research dedicated to medical genetics and somatic genomics [122]. We started to use original systems biology or bioinformatics approaches to prioritize autism/intellectual disability genes by simple protocols [123]. However, more sophisticated approaches specially elaborated for molecular cytogenetics were soon found to be required. As a result, a new dimension (in silico dimension) in molecular cytogenetics was discovered [124]. In silico molecular cytogenetics was shown to uncover molecular and cellular mechanisms of diseases associated with chromosomal imbalances and copy number variations. Additionally, it became a basis for original algorithms of fusion and network-based classification of molecular cytogenetic data [125] as well as proposing global pathways of neuropsychiatric diseases for therapeutic interventions [126]. These algorithms were successfully used for uncovering mechanisms of chromosomal instability and somatic mosaicism [127, 128]. Moreover, in silico approaches to chromosomal imbalances are applicable for developing successful treatments of chromosomal abnormalities, which are considered incurable genetic conditions [129]. To this end, we recently proposed a practical in silico protocol for molecular cytogenetic diagnosis of neuropsychiatric diseases [130]. As one can see, Yuri made a significant input in clinical (genomic) bioinformatics. His efforts in this field resulted in significant quality of life increase (or even healing) of children suffering from presumably hopeless genetic diseases.

Yuri’s contribution to practical medicine or more precisely molecular diagnosis was immense. His diagnostic research was intimately related to working at laboratory of molecular cytogenetics of neuropsychiatric diseases at Veltischev Research and Clinical Institute for Pediatrics of the Pirogov Russian National Research Medical University (Institute of Pediatrics and Pediatric Surgery), Moscow, Russia. Since the late eighties, numerous reports were published about the increase of diagnostic efficiency of molecular cytogenetic techniques [21, 24, 25, 28, 49–51, 102–107, 112, 116, 117, 130, 131]. For instance, the first Russian array CGH (comparative genomic hybridization) study of a clinical population was the result of Yuri’s tremendous efforts [131, 132]. His diagnostic research was not limited to chromosome abnormalities and copy number variations. Under Yuri’s supervision, an approach to molecular diagnosis of epigenetic diseases was proposed [133]. This led to a discovery of a new epigenomic mechanism of neurodevelopmental diseases in childhood [134]. His approaches to molecular diagnosis of structural genomic variations in autism and intellectual disability including uncovering mechanisms and possible therapies were recently found highly effective [135]. Finally, it is important to mention that Yuri’s efforts resulted in elevating the educational level in the field of molecular genetic diagnosis. Our laboratories receive consistent thanks of almost all Russian-spoken specialists from all over the world because of his co-authored books [136–142], which are the only books describing real cytogenetics, molecular cytogenetics and cytogenomics in Russian.

The latest results of Yuri’s research formed a firm basis for molecular cytogenetics earning its well-deserved place in postgenomic biomedicine. These efforts resulted in a special issue of *Current Genomics*, which uncovers new realities and dimensions of molecular cytogenetics — cytogenomics or molecular cyto(post)genomics [143]. In this issue, Yuri and his colleagues reported a part of aneuploidy research in the schizophrenia brain and comorbid psychiatric disorders [94]. Additionally, our original bioinformatic techniques were shown to be truly applicable for basic and applied cytogenomics to uncover molecular, cellular, physiological and even neuropsychological mechanisms of diseases caused by chromosome imbalances [144]. Furthermore, we proposed an original cytogenomic hypothesis suggesting that human behavior might be regulated through changes in proportions of somatic mosaicism levels resulted from complex interaction between mutational burden and environment [145]. Unfortunately, the issue was published at that time, when Yuri was unable to see it (posthumously), even though its content was prepared in 2016-2017.

A very special part of Yuri’s research life was *Molecular Cytogenetics*, the journal founded in 2008 with his essential participation [146]. It all started as a joke. In 2005, during a conversation with our good friend and colleague Professor Thomas Liehr, somebody said that it is always challenging for a group of independent scientists (i.e. researchers uninvolved in large sophisticated collaborative webs and hierarchical relationships) to publish an article containing bold ideas, unique, albeit logic and scientific, views, and own conclusions in their original form, especially in the field of cytogenetics. Jokingly, it was said that there is an easy solution to this problem: one just had to establish a new journal dedicated to chromosome biology and molecular cytogenetics based on a principle, something like “*good music for good people*” with the only exception of being a scientific journal. It is well-known that “*there is a grain of truth in every joke*”. It was a solution, but not an easy one. It took three years to begin the journal. Yuri became one of the Editors-in-Chief as one of the founders. From that time onwards it took then five years to receive the first official impact factor [147]. Further success was achieved when the journal was ranked first out of all journals specifically dedicated to cytogenetics. The editorship of *Molecular Cytogenetics* was Yuri’s honorable duty. We are only starting to understand the extent of his contribution to the journal’s success.

Yuri left endless amount of unfinished works, numerous descriptions of his original ideas and theories, and tens of thousands of paper sheets containing data. Consequently, it is now our duty to finalize the studies and to publish the results of his enormous research activity. We do hope that these publications will appear in the nearest future.

It is not a secret that Yuri’s family is the core of team members of three laboratories which have performed such a great body of biomedical research. He is a grandfather of three granddaughters, who remember him as the kindest and nicest person in the world. Thus, this is also a grievous personal loss for us, his family.

We miss him a lot! The gap in our life resulted from this untimely and unacceptable loss cannot be filled. We would like to express our thanks to all the friends and colleagues for their kind support. The only thing we can do now is to multiply and to share the legacy of such an outstanding researcher as Professor Yuri B. Yurov.
